# Validation of a deep-learning-based retinal biomarker (Reti-CVD) in the prediction of cardiovascular disease: data from UK Biobank

**DOI:** 10.1186/s12916-022-02684-8

**Published:** 2023-01-24

**Authors:** Rachel Marjorie Wei Wen Tseng, Tyler Hyungtaek Rim, Eduard Shantsila, Joseph K. Yi, Sungha Park, Sung Soo Kim, Chan Joo Lee, Sahil Thakur, Simon Nusinovici, Qingsheng Peng, Hyeonmin Kim, Geunyoung Lee, Marco Yu, Yih-Chung Tham, Ameet Bakhai, Paul Leeson, Gregory Y.H. Lip, Tien Yin Wong, Ching-Yu Cheng

**Affiliations:** 1 Singapore Eye Research Institute, Singapore National Eye Centre, Singapore, Singapore; 2 Duke-NUS Medical School, Singapore, Singapore; 3 Ophthalmology and Visual Sciences Academic Clinical Program (Eye ACP), Duke-NUS Medical School, Singapore, Singapore; 4 Mediwhale Inc., Seoul, South Korea; 5 Department of Primary Care and Mental Health, University of Liverpool, Liverpool, UK; 6 Albert Einstein College of Medicine, New York, NY USA; 7 Division of Cardiology, Severance Cardiovascular Hospital, Yonsei University College of Medicine, Seoul, South Korea; 8 Division of Retina, Severance Eye Hospital, Yonsei University College of Medicine, Seoul, South Korea; 9 Clinical and Translational Sciences Program, Duke-NUS Medical School, Singapore, Singapore; 10 Center for Innovation and Precision Eye Health, Yong Loo Lin School of Medicine, National University of Singapore, Singapore, Singapore; 11 Department of Ophthalmology, Yong Loo Lin School of Medicine, National University of Singapore, Singapore, Singapore; 12 Royal Free Hospital London NHS Foundation Trust, London, UK; 13 Cardiology Department, Barnet General Hospital, Thames House, Enfield, UK; 14 Cardiovascular Clinical Research Facility, RDM Division of Cardiovascular Medicine, University of Oxford, Oxford, UK; 15 Liverpool Centre for Cardiovascular Science, University of Liverpool and Liverpool John Moores University and Liverpool Heart & Chest Hospital, Liverpool, United Kingdom; and Department of Clinical Medicine, Aalborg University, Aalborg, Denmark; 16 Tsinghua Medicine, Tsinghua University, Beijing, China

**Keywords:** Artificial intelligence, Cardiovascular disease, Deep learning, Retinal imaging, Retinal photograph, Risk stratification, Risk stratification system, UK Biobank

## Abstract

**Background:**

Currently in the United Kingdom, cardiovascular disease (CVD) risk assessment is based on the QRISK3 score, in which 10% 10-year CVD risk indicates clinical intervention. However, this benchmark has limited efficacy in clinical practice and the need for a more simple, non-invasive risk stratification tool is necessary. Retinal photography is becoming increasingly acceptable as a non-invasive imaging tool for CVD. Previously, we developed a novel CVD risk stratification system based on retinal photographs predicting future CVD risk. This study aims to further validate our biomarker, Reti-CVD, (1) to detect risk group of ≥ 10% in 10-year CVD risk and (2) enhance risk assessment in individuals with QRISK3 of 7.5–10% (termed as borderline-QRISK3 group) using the UK Biobank.

**Methods:**

Reti-CVD scores were calculated and stratified into three risk groups based on optimized cut-off values from the UK Biobank. We used Cox proportional-hazards models to evaluate the ability of Reti-CVD to predict CVD events in the general population. C-statistics was used to assess the prognostic value of adding Reti-CVD to QRISK3 in borderline-QRISK3 group and three vulnerable subgroups.

**Results:**

Among 48,260 participants with no history of CVD, 6.3% had CVD events during the 11-year follow-up. Reti-CVD was associated with an increased risk of CVD (adjusted hazard ratio [HR] 1.41; 95% confidence interval [CI], 1.30–1.52) with a 13.1% (95% CI, 11.7–14.6%) 10-year CVD risk in Reti-CVD-high-risk group. The 10-year CVD risk of the borderline-QRISK3 group was greater than 10% in Reti-CVD-high-risk group (11.5% in non-statin cohort [*n* = 45,473], 11.5% in stage 1 hypertension cohort [*n* = 11,966], and 14.2% in middle-aged cohort [*n* = 38,941]). *C* statistics increased by 0.014 (0.010–0.017) in non-statin cohort, 0.013 (0.007–0.019) in stage 1 hypertension cohort, and 0.023 (0.018–0.029) in middle-aged cohort for CVD event prediction after adding Reti-CVD to QRISK3.

**Conclusions:**

Reti-CVD has the potential to identify individuals with ≥ 10% 10-year CVD risk who are likely to benefit from earlier preventative CVD interventions. For borderline-QRISK3 individuals with 10-year CVD risk between 7.5 and 10%, Reti-CVD could be used as a risk enhancer tool to help improve discernment accuracy, especially in adult groups that may be pre-disposed to CVD.

**Supplementary Information:**

The online version contains supplementary material available at 10.1186/s12916-022-02684-8.

## Background

In the United Kingdom (UK), around 1 in 9 people live with cardiovascular disease (CVD) [1, 2]. A formal risk assessment tool, QRISK3, was developed using data from a cohort of 1.28 million individuals, and it is currently recommended by the National Institute for Health and Care Excellence (NICE) for CVD risk assessment and guidance for primary prevention strategies [3]. In 2014, NICE updated the requirements for statin initiation in individuals at risk of developing CVD, increasing the number of eligible individuals for statin treatment [4]. NICE also updated the requirements for antihypertensive initiation in individuals with stage 1 hypertension (clinically-measured blood pressure ranging from 140/90 to 159/99 mmHg) in 2019 [5]. The current recommendation for initiating statins for primary CVD prevention and antihypertensives for stage 1 hypertension is a calculated QRISK3 score of 10% [4, 5]. Therefore, the benchmark of 10% for 10-year CVD risk is very important in determining clinical intervention.

Despite these updated guidelines, their effect on clinical practice has been limited, with little change in statin prescription behavior [6]. Moreover, considering that 10-year CVD risk might underestimate the lifetime probability of developing CVD, NICE recommends that antihypertensive treatment should also be considered for people under 60 who have a predicted 10-year CVD risk below 10%. Practice-changing guidelines would require time-consuming longitudinal prospective studies. A simple and personalized CVD risk refinement tool may be helpful in not only minimizing time and various resource costs, but also finding consensus on statin and antihypertensive treatment initiation.

Retinal photography is a simple, effective, and non-invasive imaging tool that provides fast and accurate information on the human vasculature that may not be clearly visible to the human eye especially in recent years with the advent of deep learning (DL) [7–9]. In our previous study, we developed a DL-based three-tier CVD risk stratification system, RetiCAC, using coronary artery calcium (CAC) as a ground truth, which allowed us to stratify CVD risk in the UK Biobank and a diversified Asian population [10]. However, this study had no comparison of RetiCAC with QRISK3 and was not UK-specific, thereby limiting its application in clinical decision-making such as statin and anti-hypertensive medication initiation in the UK.

In this study, we aimed to optimize the operating threshold of RetiCAC, herein referred to as Reti-CVD, via simple retinal imaging. Using Reti-CVD, we aimed (1) to detect risk group of ≥ 10% in 10-year CVD risk and (2) to evaluate the potential of Reti-CVD as a risk enhancer when applied alongside QRISK3 for borderline-QRISK3 groups (i.e., individuals with a 10-year CVD risk of 7.5–10%) in the UK Biobank.

## Methods

### Ethics statement

This retrospective study was deemed exempt from institutional review board (IRB) review by the SingHealth Centralised Institutional Review Board (CIRB). This study adhered to the tenets of the Declaration of Helsinki. Written informed consent was obtained from the participants of the original studies [10, 11].

### Study population

We used clinical data and retinal photographs from the UK Biobank, a prospective population-based cohort in the UK [12]. The UK Biobank protocol is available online [13].

We excluded (1) duplicated retinal photographs (*n* = 18,423), (2) those who had type 1 diabetes (*n* = 288), (3) those with pre-existing CVD at baseline (*n* = 7624), (4) poor-quality photographs (*n* = 11,115), and (5) those who were < 40 years old (*n* = 1) (Additional file 1: eFigure 1). Pre-existing CVD was defined as previous history of coronary heart disease, other heart diseases, stroke, transient ischemic attack, peripheral arterial disease, or cardiovascular surgery, and patients who have undergone cardiovascular procedures based on the Classification of Interventions and Procedures version 4 (OPCS-4) [10].

A total of 48,260 participants, representing the general population without a history of CVD, were included for analysis. In addition, we also defined three at-risk subgroups: (1) the non-statin cohort, individuals not taking a statin; (2) the stage 1 hypertension cohort, individuals with stage 1 hypertension and not taking any antihypertensive medications; and (3) the middle-aged cohort, individuals aged 40–64 years at baseline (Additional file 1: eFigure 1). Since the purpose of Reti-CVD is aimed at primary prevention of CVD, we focus on individuals who display a lack of awareness towards the risk factors associated with cardiovascular disease. In a previous publication, the authors reported based on age-stratified analyses that unawareness rates were highest in individuals aged between 40 and 49 years old and lowest in individuals aged above 70 years old [14]. Therefore, our middle-aged cohort is classified based on vulnerability.

Retinal photographs included in the study were taken using the Topcon 3D OCT-1000 Mark II (Topcon Corporation) between 7 December 2009 and 21 July 2010. Retinal cameras used in the training set include AFP-210 non-mydriatic auto retinal camera (NIDEK Corporation, Aichi, Japan), TRC-NW8 non-mydriatic retinal camera (Topcon Corporation, Tokyo, Japan), and Nonmyd A-D (Kowa Co. Ltd., Shizuoka, Japan). We did not include Topcon 3D OCT-1000 Mark II (Topcon Corporation) in our training set.

The other variables used in this study were defined as follows. Pre-diabetes and diabetes were defined based on (1) medical history and (2) glucose ≥ 5.5 mmol/L. Medical history of high cholesterol, type 1 diabetes, antihypertensive were self-declared and collected from a baseline assessment questionnaire on medical conditions. Smoking status was self-declared as well and categorized into “life-time smoker” and “never.”

### Definition of cardiovascular disease events

In the UK Biobank, we used hospitalization and mortality data provided by the National Health Service (NHS) registers. The main outcome of interest in the current study reflected the outcome used in the QRISK3 risk score: fatal CVD events (ICD-10 I00-99, F01, Q20-Q28, C38.0, P29, G45) [15] or nonfatal coronary heart disease, ischemic stroke, or transient ischemic attack (ICD-10 G45, I20–24, and I63–64) [16].

### Calculation of the QRISK3 score and borderline-QRISK3 group

For each individual, the QRISK3 score was calculated using R package, version 3.6 [17]. The distribution of the QRISK3 score is provided in Additional file 2: eFigure 2. For comparison with three-strata Reti-CVD groups, we divided the subjects into 5 groups based on the QRISK3 score (%) (≥ 0 to < 5; ≥ 5 to > 10; ≥ 10 to > 15; ≥ 15 to < 20; and ≥ 20). In addition, as the recommended threshold for initiating statins and antihypertensive medication from a 10-year risk of CVD is 10%, we defined “borderline-risk” group as those who had QRISK3 score between 7.5 and 10%. Specifically, we divided the subjects into 5 groups as compared to the previous three-risk-strata group of QRISK3 as outlined in the NICE guidelines. Herein lies the difference in intervals used to evaluate CVD events. We used 5% intervals compared to the previous 10% intervals so that a more detailed comparison could be done with Reti-CVD.

### New retinal-based cardiovascular disease risk stratification system ≈

Details of RetiCAC model development and previous validations have been described elsewhere [9]. Briefly, the RetiCAC score was defined based on a probability score derived from our deep-learning algorithm of binary classification (absence vs presence of coronary artery calcium [CAC]). The probability scores ranged from zero to one, with a high value indicating a high probability of the presence of CAC. First, we enhanced the RetiCAC algorithm using more datasets which includes both retinal photographs and CT scans from Korea (Additional file 3: eDocument 1). Second, we proposed new cardiovascular disease risk stratification groups (i.e., Reti-CVD) with optimized cut-off values based on 40th percentile and 95th percentile of Reti-CVD score among 48,260 participants after exclusion. The cut-off values were determined to have similar incidence rate: the low-risk group of Reti-CVD was designed to have similar incidence rate to those of the 0 to 5% QRISK3 risk group (i.e., 2.5 per 1000 person-years), and the moderate-risk of Reti-CVD group was designed to have similar incidence rate to those of the 5 to 10% QRISK3 risk group (i.e., 7.0 per 1000 person-years). We used these proposed cut-off values to further stratify the CVD risk in the UK Biobank participants.

### Statistical analysis

Analyses were done using *p* < 0.05 as the significance level, Stata/MP version 14.0 for survival analysis, and R version 3.4.4 for estimation of net reclassification index (NRI) using the R package survIDINRI [18]. Descriptive statistics are provided for all participants and also according to the three risk groups by Reti-CVD.

In the UK Biobank, hospitalization and mortality data were available up to May 05, 2021, at the time of analysis and each participant was followed up to 11.4 years from the date of baseline visit. In survival analysis, each patient was followed up to 11.4 years (median follow-up, 11.0 years) from the date of baseline visit to the last follow-up date or the date of the CVD events.

In all populations, the cumulative incidence of cardiovascular events rate was evaluated across the three groups (low, moderate, and high risk) defined by the Reti-CVD using Kaplan-Meier method. Cox proportional hazards model was used to estimate the hazard ratios (HRs) and trends in HRs and respective *p*-values were examined by fitting a linear model for the three categories. Unadjusted HR was provided according to three risk groups of Reti-CVD, and QRISK3-ajudted HR trend was provided.

To help future patients make an informed decision on statin and antihypertensive medication initiation, we only included the borderline-QRISK3 group who had QRISK3 score between 7.5 and 10% of 10-year CVD risk. In the borderline QRISK3 group, cumulative incidence of cardiovascular events rate was evaluated across the three groups (low, moderate, and high risk) according to the Reti-CVD and compared with participants with QRISK3 5–7.5% and 10–12.5%. The same analysis was repeated for middle-aged group (40 to 64 years).

The incremental prognostic value of the Reti-CVD over the QRISK3 in the prediction of CVD events was assessed using C-statistics and continuous net reclassification index (NRI) [18]. In addition, decision curve analysis was used to compare the net-benefit of models at different thresholds over QRISK3 model and Reti-CVD plus age and gender model. Age and gender were included for fair comparison because QRISK3 is based on survival model including various risk factors including age, gender, smoking, and comorbidities. Continuous models were presented as decision-analysis curves (demonstrating potential outcomes of using any threshold in that model) and models with higher net-benefit were considered higher performing.

## Results

### Study population characteristics

Table 1 details the clinical characteristics of the participants by the three groups of Reti-CVD score (categorized as low, moderate, and high-risk groups).

**Table 1:** Characteristics of the study population

	Reti-CVD score		
Characteristics	Low risk	Moderate risk	High risk
No. of participants	19,304	26,543	2413
**Cardiovascular outcomes**			
Nonfatal and fatal CVD events (QRISK)	545 (2.8%)	1900 (7.2%)	321 (13.3%)
**QRISK3**			
QRISK3 score, mean (SD)	3.0 (2.5)	7.0 (4.1)	10.6 (4.6)
QRISK3 score			
≥ 0 to < 5	16,282 (84.3%)	9646 (36.3%)	208 (8.6%)
≥ 5 to < 10	2572 (13.3%)	11,393 (42.9%)	967 (40.1%)
≥ 10 to < 15	384 (2.0%)	4408 (16.6%)	876 (36.3%)
≥ 15 to < 20	55 (0.3%)	880 (3.3%)	272 (11.3%)
> 20	11 (0.1%)	216 (0.8%)	90 (3.7%)
**Clinical biomarkers**			
Age, mean (SD)	50.8 (7.0)	59.8 (6.7)	64.4 (4.5)
Gender			
Female, *n* (%)	12,702 (65.8%)	13,622 (51.3%)	741 (30.7%)
Male, *n* (%)	6602 (34.2%)	12,921 (48.7%)	1672 (69.3%)
**Other factors**			
Antihypertensive medication, *n* (%)	1637 (8.5%)	5642 (21.3%)	846 (35.1%)
Stage 1 hypertension, *n* (%)	4199 (21.8%)	7178 (27.0%)	589 (24.4%)
Pre-diabetes and diabetes, *n* (%)	269 (1.4%)	677 (2.6%)	86 (3.6%)
Statin, *n* (%)	694 (3.6%)	1923 (7.2%)	170 (7.0%)
Current smoker, *n* (%)	6451 (33.4%)	11,221 (42.3%)	1219 (50.5%)

Data are presented as *n*, *n* (% of participants), mean (standard deviation [SD]), or median (interquartile range [IQR]). *CVD* cardiovascular disease. *Reti-CVD* deep-learning-based retinal CVD biomarker

Among 48,260 included participants from the UK Biobank, the median QRISK3 10-year CVD risk was 4.5% (IQR 2.2–8.0%, SD, 4.2%); CVD events occurred in 2766 (5.7%) participants during the follow-up. The Spearman’s rank correlation coefficient between Reti-CVD and QRISK3 score was 0.50 (*p* < 0.001).

CVD events rate were 2.8% (545/19,304) in the low-risk group, 7.2% (1900/26,543) in the moderate-risk group, and 13.3% (321/2413) in the high-risk group of Reti-CVD. Among the low-risk group of Reti-CVD, 84.3% had QRISK3 score of 0 to < 5%, and among the high-risk group of Reti-CVD, 8.6% had QRISK3 score of 0 to 5%, 40.1% had QRISK3 score of ≥ 5 to < 10%, and 36.3% had QRISK3 score of ≥ 10 to < 15%.

### Performance of Reti-CVD in prediction of CVD event

**Fig. 1:**
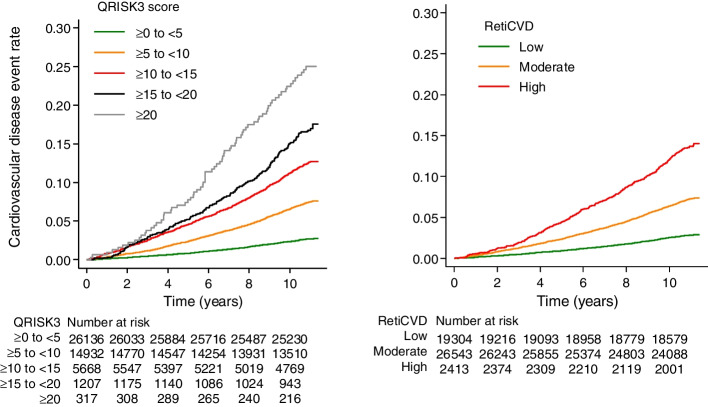
Kaplan-Meier curves according to Reti-CVD and QRISK3. Cardiovascular disease (CVD) event rate according to QRISK3-five groups (**A**), and Reti-CVD-three groups (**B**) in all participants (*n* = 48,260) were presented. In the general population, the high-risk group of Reti-CVD (**B**) shows a similar incident CVD rate to that of the QRISK 10–15% group (**A**), indicating that if a participant is identified as Reti-CVD-high risk by retinal photography, participants may be advised to undergo a full CVD risk assessment via the NHS primary care service

Kaplan-Meier curves were described in all participants according to five groups of QRISK3 and three groups of Reti-CVD (Fig. 1). During the follow-up (median 11.0 years; interquartile range [IQR], 10.9–11.1 years), 513,714.3 person-years were analyzed. As expected, QRISK3 stratified CVD risk well in general population of the UK Biobank (Fig. 1A). Reti-CVD also shows distinct CVD risk stratification based on the three groups within the general population of the UK Biobank (Fig. 1B). Based on Reti-CVD, the incidence of CVD per 1000 person-years was 2.6 (95% CI, 2.4–2.8) in the low-risk group, 6.8 (6.5–7.1) in the moderate-risk group, and 13.1 (11.7–14.6) in the high-risk group (Table 2), indicating a 13.1% 10-year CVD risk in Reti-CVD-high-risk group. Analysis for non-statin cohort and stage 1 hypertension cohort are provided in Additional file 4: eFigure 3 and Additional file 5: eTable 1.

**Table 2:** Risk of cardiovascular events by the deep-learning-based retinal CVD biomarker (Reti-CVD) in all participants

Risk predictor	*N*	Cases	Person-years	Incidence (95% CI)	Unadjusted hazard ratio (95% CI)
Reti-CVD					
Low	19,304	545	209,186	2.6 (2.4–2.8)	1 (reference)
Moderate	26,543	1900	280,021	6.8 (6.5–7.1)	2.62 (2.38–2.88)
High	2413	321	24,508	13.1 (11.7–14.6)	5.11 (4.45–5.86)
					Adjusted HR trend^a^
					1.41 (1.30–1.52)
QRISK3 score					
≥ 0 to < 5	26,136	702	283,604	2.5 (2.3–2.7)	1 (reference)
≥ 5 to < 10	14,932	1099	157,442	7.0 (6.6–7.4)	2.84 (2.58–3.12)
≥ 10 to < 15	5668	695	57,723	12.0 (11.2–13.0)	4.93 (4.44–5.48)
≥ 15 to < 20	1207	196	11,988	16.3 (14.2–18.8)	6.75 (5.76–7.91)
≥ 20	317	74	2957	25.0 (19.9–31.4)	10.50 (8.27–13.35)
Total	48,260	2766	513,714	5.4 (5.2–5.6)	

^a^Based on multivariable model after adjusting QRISK3 five groups. Incidence per 1000 person-years. *CI* confidence interval. *CVD* cardiovascular disease. *HR* hazard ratio. *N* number at risk. *Reti-CVD* deep-learning-based retinal CVD biomarker

Additional file 6: eTable 2 describes the performance of Reti-CVD in predicting the incidence of CVD events when applied to at-risk subgroup populations. Incidence of CVD and QRISK3-adjusted HR trends were provided in 3 subgroups: individuals with body-mass index (BMI) above 25 kg/m^2^, hypertension, and pre-diabetes/diabetes. Our results show that the three-strata Reti-CVD system can further stratify CVD risk in all at-risk subgroup populations. Specifically, in the subgroup of individuals who were taking antihypertensive medication, the incidence of CVD was as high as 17.7 (95% CI, 15.0–20.8) in Reti-CVD-high-risk group.

### Performance of Reti-CVD as a risk enhancer in borderline-QRISK3 group

**Fig. 2:**
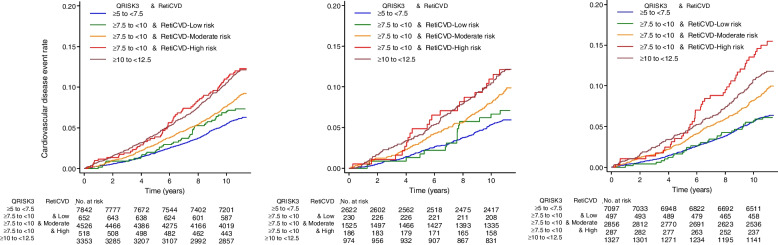
Kaplan-Meier curves according to Reti-CVD in borderline-QRISK3 group. Cardiovascular disease (CVD) event rate according to Reti-CVD-three groups in borderline-QRISK3 group who had QRISK3 score between 7.5 and 10% were presented in non-statin cohort (**A**), stage 1 hypertension cohort (**B**), and middle-aged (40–64 years) cohorts (**C**). Considering that statin and antihypertensive pharmacotherapy initiation is recommended at QRISK of ≥ 10%, Reti-CVD can be used as a risk enhancer in borderline-QRISK3 groups of 7.5–10% to reach consensus on statin initiation. In addition, although most of risk assessment systems were derived from cohorts of primarily middle-aged people and typically well-functioning individuals, Reti-CVD can still be a risk enhancer in in borderline-QRISK3 groups of 7.5–10% middle-aged people

The Kaplan-Meier analysis of CVD events in the UK Biobank according to the three strata of Reti-CVD among borderline group individuals (QRISK3 score between 7.5 and 10%) is provided in Fig. 2. Although QRISK3 was less than 10% in the borderline group, the cumulative CVD events rate in Reti-CVD-high-risk group was consistently higher than 10% of 10-year CVD risk.

Specifically, in this borderline-QRISK3 group, the 10-year CVD risk was higher than 10% in Reti-CVD-high-risk group with 11.5% (8.9–15.0%) in non-statin cohort (*n* = 45,473), 11.5% (7.4–17.8%) in stage 1 hypertension cohort (*n* = 11,966), and 14.2% (10.3–19.5%) in middle-age cohort (*n* = 38,941) (Table 3).

**Table 3:** Risk of cardiovascular events by the deep-learning-based retinal CVD biomarker (Reti-CVD) in participants who had 7.5–10% QRISK3 score

QRISK3	Reti-CVD^a^	*N*	Cases	Person-years	Incidence rate at year 10 (95% CI)
**Non-statin cohort (*n*** **= 45,473)**					
≥ 5 to < 7.5		7842	478	83,295	5.6 (5.1–6.1)
≥ 7.5 to < 10	Low	652	47	6868	7.2 (5.4–9.6)
≥ 7.5 to < 10	Moderate	4526	401	47,286	7.9 (7.1–8.8)
≥ 7.5 to < 10	High	518	62	5318	11.5 (8.9–15.0)
≥ 10 to < 12.5		3353	394	34,314	11.0 (9.9–12.2)
Total		16,891	1382	177,081	7.8 (7.4–8.2)
**Stage 1 Hypertension cohort (*n*** **= 11,966)**					
≥ 5 to < 7.5		2622	154	27,891	5.5 (4.6–6.5)
≥ 7.5 to < 10	Low	230	16	2427	6.8 (4.1–11.3)
≥ 7.5 to < 10	Moderate	1525	143	15,821	8.3 (6.9–9.9)
≥ 7.5 to < 10	High	186	22	1903	11.5 (7.4–17.8)
≥ 10 to <1 2.5		974	115	9990	10.9 (9.0–13.3)
Total		5537	450	58,032	7.8 (7.1–8.5)
**Middle**–**aged cohort (40 to 64 years,** ***n*** **= 38,941)**					
≥ 5 to < 7.5		7097	442	75,354	5.7 (5.2–6.3)
≥ 7.5 to < 10	Low	497	30	5291	5.8 (4.0–8.4)
≥ 7.5 to < 10	Moderate	2856	272	29,813	8.5 (7.5–9.7)
≥ 7.5 to < 10	High	287	43	2914	14.2 (10.3–19.5)
≥ 10 to < 12.5		1327	153	13,658	11.2 (9.5–13.2)
Total		12,064	940	127,030	7.4 (6.9–7.9)

^a^Reti-CVD only applied on borderline-QRISK3 groups. Incidence per 1000 person-years at year 10. *CI* confidence interval. *N* number at risk. *Reti-CVD* deep-learning-based retinal CVD biomarker

### Risk discrimination and incremental value

We then tested the incremental value of Reti-CVD when applied alongside QRISK3 in predicting incident CVD events among non-statin, stage 1 hypertension, and middle-aged cohorts (Table 4). By adding Reti-CVD score to QRISK3, *C* statistics increased by 0.014 (95% CI, 0.010–0.017) in non-statin cohort, 0.013 (0.007–0.019) in stage 1 hypertension cohort, and 0.023 (0.018–0.029) in middle-aged cohort for prediction of CVD events. Also, by adding Reti-CVD score to QRISK3, the continuous NRI was 0.133 (95% CI, 0.088–0.173) in non-statin cohort, 0.094 (0.008–0.174) in stage 1 hypertension cohort, and 0.248 (0.190–0.301) in middle-aged cohort.

**Table 4:** Prognostic performance with the addition of Reti-CVD to the QRISK3 in the UK biobank dataset

	Non-statin cohort (*n* = 42,473)		Stage 1 hypertension cohort (*n* = 11,966)		Middle-aged Cohort (*n* = 38,941)	
Models	*C* statistic (95% CI)	*p* value	*C* statistic (95% CI)	*p* value	*C* statistic (95% CI)	*p* value
Reti-CVD	0.624 (0.615–0.633)	NA	0.591 (0.575–0.608)	NA	0.611 (0.600 -0.622 )	NA
Reti-CVD plus age, gender	0.699 (0.690–0.709)	NA	0.663 (0.644–0.681)	NA	0.681 (0.669–0.692)	NA
QRISK3	0.682 (0.672–0.692)	NA	0.639 (0.620–0.658)	NA	0.650 (0.638–0.662)	NA
Reti-CVD plus QRISK3	0.696 (0.686–0.706)	NA	0.652 (0.633–0.671)	NA	0.674 (0.661–0.686)	NA
Δ Reti-CVD plus QRISK3 versus QRISK3	0.014 (0.010–0.017)	< 0.001	0.013 (0.007–0.019)	< 0.001	0.023 (0.018–0.029)	< 0.001
NRI						
Continuous NRI (95% CI)	0.133 (0.088–0.173)	< 0.001	0.094 (0.008–0.174)	0.033	0.248 (0.190–0.301)	< 0.001

Data are *C* statistic (95% CI), unless stated otherwise. The Reti-CVD plus QRISK3 model is a logistic model fit on the UK Biobank. NRI for Reti-CVD plus QRISK3 versus QRISK3 models was provided

*NRI* net reclassification index, *CI* confidence interval, *CVD* cardiovascular disease, *Reti-CVD* deep-learning-based retinal CVD biomarker

Additional file 7: eFigure 4 summarizes net-benefit comparisons using a decision-analysis curve. In entire UK Biobank participants, Additional file 7: eFigure 4A presents the decision-analysis curves for QRISK3 score and Reti-CVD plus age, and gender model, indicating that the two models are almost similar in terms of net-benefit. In the hypertensive patients and the pre-diabetes and diabetes subgroup (Additional file 7: eFigure 4B and 4C), the Reti-CVD plus age, and gender model demonstrated a higher net-benefit than QRISK3 score in general.

## Discussion

In this study, we optimized the operating threshold of RetiCAC, which was originally based on the Korean cohort. This optimized biomarker, Reti-CVD, stratified the general UK population into three risk groups and presented with significant hazard ratio (HR) trends. Specifically, the high-risk group in Reti-CVD adequately identified individuals who had a 10-year CVD risk greater than 10%, with a CVD incidence of 13.1 per 1000 person-years. Moreover, we quantified the cumulative CVD events and prognostic value for risk stratification when adding Reti-CVD alongside QRISK3 in the borderline-QRISK3 group (10-year CVD risk of 7.5–10%). Due to the new cut-off threshold of 10% for statin and antihypertensive treatment initiation, we focused on individuals who had a QRISK3 score between 7.5 and 10% and showed that Reti-CVD could further stratify the CVD risk into low, moderate, and high-risk groups. Specifically, in the borderline-risk group, individuals who were stratified as high-risk using Reti-CVD had a 10-year CVD risk greater than 10% and was comparable to the CVD incidence of QRISK3 (10 to 12.5%) group, indicating that Reti-CVD could be used as a risk enhancer.

Like other areas of medicine, there is now a paradigm shift in cardiovascular medicine, towards using digital innovations especially those related to artificial intelligence to enhance diagnosis and risk stratification [19]. Retinal imaging has been posited as a potential imaging application for risk assessment in a wide variety of diseases. Reti-CVD, as a further extension of RetiCAC, when applied alongside QRISK3, provides a good reflection of CVD severity in the UK population. This underscores the importance of the retinal imaging-based Reti-CVD tool for clinical CVD risk stratification.

Previous studies using retinal imaging as a tool for predicting CVD risk have focused largely on other risk factors such as age, hypertension, and dyslipidemia [20–22]. Other studies have investigated the use of retinal imaging to determine the risk of specific CVD subtypes such as coronary heart disease [23] and peripheral arterial disease [24]. Compared to these studies, our study focuses on predicting future CVD events in general instead of diagnosis of current subtypes of CVD. Studies that have used DL-based retinal imaging to predict coronary artery calcium (CAC) scores were either applied on small sample sizes and/or did not evaluate the ability of the deep learning systems to predict future CVD events [25, 26]. In particular, a study by Chang et al. evaluated the value-added ability of its DL-based system in predicting CVD mortality from retinal imaging when added to the Framingham risk scoring (FRS) system [27]. However, Hippisley-Cox et al has suggested that the FRS algorithm has ethnicity bias and may be less suited to the UK cohort compared to QRISK algorithms [3]. Therefore, our novel focus in applying Reti-CVD to QRISK3 reflects both the UK Biobank demographic population and NICE guidelines, as QRISK3 was recommended by NICE.

In the general population, we have shown that Reti-CVD can identify individuals with a CVD risk greater than 10% in our high-risk Reti-CVD group. This finding is useful for two reasons because a CVD risk of 10% is the latest threshold for statin and anti-hypertensive medication initiation. First, at the primary care level, Reti-CVD provides GPs with a simple and effective way of providing an initial assessment on asymptomatic patients. Ophthalmologists, optometrists, and opticians can also use existing retinal photographs for opportunistic eye screening. Second, Reti-CVD can aid the CVD prevention program that NHS England has developed. The NHS CVD primary prevention focusses on three main components: atrial fibrillation, blood pressure, and cholesterol [28]. Given that our study included individuals who are not on statin or anti-hypertensive medication in the general population, physicians can use Reti-CVD while monitoring patients’ blood pressure and cholesterol levels to better predict CVD risk. If identified as high-risk by Reti-CVD, patients can be encouraged to undergo a NHS health check-up for further evaluation. This could boost the compliance rate for NHS health screening, which has been estimated to be less than 50% [29]. This low-cost opportunistic CVD risk screening based on routine eye checks may help to include ‘hard to reach' populations in CVD risk assessment.

An additional finding that we want to highlight is the fact that Reti-CVD identified high-risk individuals who were otherwise missed by QRISK3. 8.6% of patients identified as high-risk by our new stratification tool had a QRISK3 score between 0 to 5 and 40.1% of them were categorized in the QRISK3 ≥ 5 to < 10% group. This demonstrates the potential of Reti-CVD as an adjunct tool to identify future high-risk individuals who may be left out if CVD risk stratification is dependent solely on QRISK3 scoring. Consequently, when we incorporate only two basic demographic factors of age and gender to Reti-CVD, this model was superior to QRISK3 model across the range of risk thresholds. Moreover, in our explorative analysis in eTable 3, we confirmed that upon conducting a more detailed QRISK3 analysis in the borderline QRISK3 group where individuals were divided based on 0.5 intervals between 7.5 and 10%, more than 1.6% of individuals were classified in the Reti-CVD-high-risk groups in all subgroups. This indicates that Reti-CVD can be used as an independent risk enhancer in this range of QRISK3.

By comparing Reti-CVD to QRISK3, this study demonstrates the potential value and scope of Reti-CVD to improve discernment accuracy in the UK population since QRISK3 is part of the up-to-date guidelines that reflects the demographic profile of the UK Biobank. Study limitations include a possibility of systematic errors in the form of misclassification bias, since hospitalization and mortality data provided by the NHS registers were used for CVD definition. However, a study by Kivimaki et al. has analyzed the validity of and reported good agreement between CVD event predictions and the use of UK National Health Electronic Records, therefore minimizing the likelihood of CVD event misdiagnoses [30]. We also excluded poor quality images during the development and validation of Reti-CVD. The quality of retinal photographs available in clinical settings may vary, and therefore this may pose challenges for real-world implementation.

## Conclusions

In conclusion, a DL and retinal photograph-derived new CVD biomarker, Reti-CVD, could effectively stratify CVD risk in the general population. We also confirmed that Reti-CVD has the potential to identify individuals with ≥ 10% 10-year CVD risk who are likely to benefit from preventative CVD interventions. In addition, for people with a QRISK3 score between 7.5 and 10%, Reti-CVD could be used as a risk stratification enhancer for identifying high risk patients for aggressive CVD prevention.

## Supplementary Information

**Additional file 1: eFigure 1.** Study flow chart.

**Additional file 2: eFigure 2.** Distribution of the QRISK3 score in the UK Biobank.

**Additional file 3: eDocument 1.** RetiCAC model updates.

**Additional file 4: eFigure 3.** Kaplan-Meier curves according to Reti-CVD and QRISK3.

**Additional file 5: eTable 1.** Risk of cardiovascular events by the deep-learning-based retinal CVD biomarker (Reti-CVD).

**Additional file 6: eTable 2.** Subgroup analysis in the UK Biobank.

**Additional file 7: eFigure 4.** Decision curve analysis.

**Additional file 8: eTable 3.** Detailed distribution of Reti-CVD risk groups among those between 7.5% and 10% QRISK3 score.

## Data Availability

The UK Biobank test dataset was obtained from UK Biobank (application number 68428). Data cannot be shared publicly due to the violation of patient privacy and the absence of informed consent for data sharing.

## Abbreviations

CVD: Cardiovascular disease
UK: United Kingdom
NICE: National Institute for Health and Care Excellence
DL: Deep-learning
CAC: Coronary artery calcium
IRB: Institutional Review Board
CIRB: SingHealth Centralized Institutional Review Board
NHS: National Health Service
NRI: Net Reclassification Index
BMI: Body-mass index
HR: Hazard ratio
FRS: Framingham risk score
GP: General practitioner
