# Supplementary figures and images for: Validation of a deep-learning-based retinal biomarker (Reti-CVD) in the prediction of cardiovascular disease: data from UK Biobank
Source: BMC Med. 2023 Jan 24;21:28. doi: 10.1186/s12916-022-02684-8 (PMC9872417; doi:10.1186/s12916-022-02684-8)

Additional file 1: eFigure 1. Study flow chart


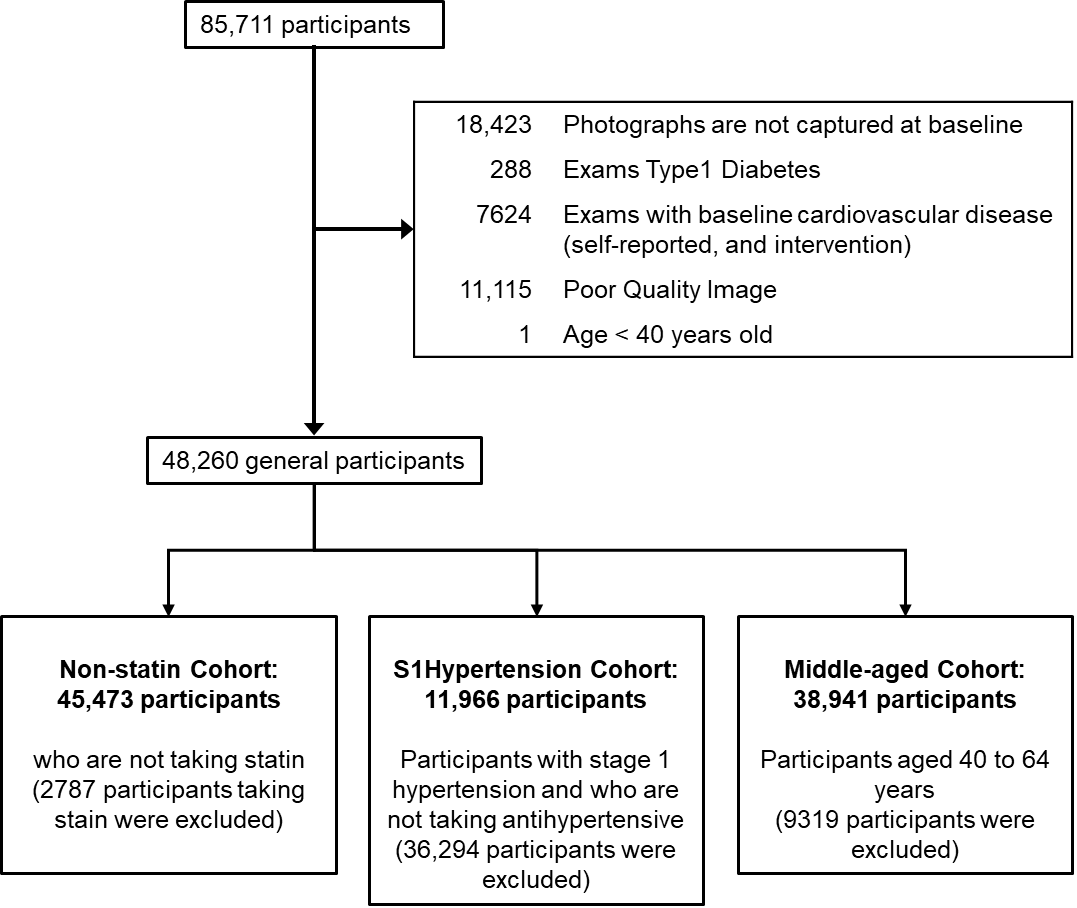

Supplement: Supplementary file 1 — Additional file 1: eFigure 1. Study flow chart. [file 12916_2022_2684_MOESM1_ESM.docx]
