# Supplementary material for: Validation of a deep-learning-based retinal biomarker (Reti-CVD) in the prediction of cardiovascular disease: data from UK Biobank
Source: BMC Med. 2023 Jan 24;21:28. doi: 10.1186/s12916-022-02684-8 (PMC9872417; doi:10.1186/s12916-022-02684-8)
Supplement: Supplementary file 2 — Additional file 2: eFigure 2. Distribution of the QRISK3 score in the UK Biobank. [file 12916_2022_2684_MOESM2_ESM.docx]

## Additional file 2: eFigure 2. Distribution of the QRISK3 score in the UK Biobank
