# Supplementary material for: Validation of a deep-learning-based retinal biomarker (Reti-CVD) in the prediction of cardiovascular disease: data from UK Biobank
Source: BMC Med. 2023 Jan 24;21:28. doi: 10.1186/s12916-022-02684-8 (PMC9872417; doi:10.1186/s12916-022-02684-8)
Supplement: Supplementary file 3 — Additional file 3: eDocument 1. RetiCAC model updates. [file 12916_2022_2684_MOESM3_ESM.docx]

## Additional file 3: eDocument 1. RetiCAC model updates

In our previous publication, we had two large health screening datasets 1 and 2 which included retinal photograph and cardiac CT scan on the same day, and we developed RetiCAC using dataset 1 previously. The performance of RetiCAC in predicting individuals with the presence of CAC was AUC of 0.731 (95% CI, 0.712-0.751) in the internal test set. In this study, we included both datasets for training RetiCAC – the same dataset used in the previous publication and an additional dataset from a second Korean screening centre. Altogether, we included 31,942 exams (63,884 photos) from 28,583 participants who took a retinal photograph and cardiac CT scan on the same day. This combined dataset was randomly distributed into a developmental set and an internal test set with an 8:2 ratio. To prevent overfitting, we divided the datasets by individuals and, therefore, there was no “crossing over” of data of the same individual across the developmental and internal test sets. We also used different deep learning architecture and training.

1) Model architecture

Our deep learning model is based on the convnext model^[[1]](#footnote-1)^ which outperformed previous CNN models. Unlike 3x3 convolutional layers were mainly adopted in previous CNN models, convnext adopted 7x7 size kernel for larger receptive fields. Our model design was almost identical to convnext except for the last fully connected layer’s dimension which was changed to 3 for 3 cut-off (CACS score >0, >100, >300) probability predictions. Logit resulted from last fully connected layer is converted to probability with sigmoid function and we trained model to minimize loss from target and prediction.

2) Training setting

We trained our model using adam optimizer with 2e-4 learning rate, cosine learning rate schedule during 25 epochs and used mixup, cutmix, randaugment, enhancing contrast module, random crop for data augmentation. Additionally, we adopted focal loss, exponential moving average and used 384x384 size images. Lastly, to relieve hard label problem which 299 CAC score is classified to not over 300, we applied soft label to 100 and 300 CACS score cut-off training using sigmoid function.

We termed this updated RetiCAC deep learning model as Reti-CVD, and Reti-CVD show higher AUC of 0.779 (95% CI, 0.768 - 0.790) in internal test set than that of previous AUC of 0.731 (95% CI, 0.712-751).

1. Liu, Zhuang, et al. "A ConvNet for the 2020s." arXiv preprint arXiv:2201.03545 (2022). [↑](#footnote-ref-1)
