# Supplementary material for: Validation of a deep-learning-based retinal biomarker (Reti-CVD) in the prediction of cardiovascular disease: data from UK Biobank
Source: BMC Med. 2023 Jan 24;21:28. doi: 10.1186/s12916-022-02684-8 (PMC9872417; doi:10.1186/s12916-022-02684-8)

## Additional file 4: eFigure 3. Kaplan-Meier curves according to Reti-CVD and QRISK3

**A QRISK3 in non-statin cohort B Reti-CVD in non-statin cohort**


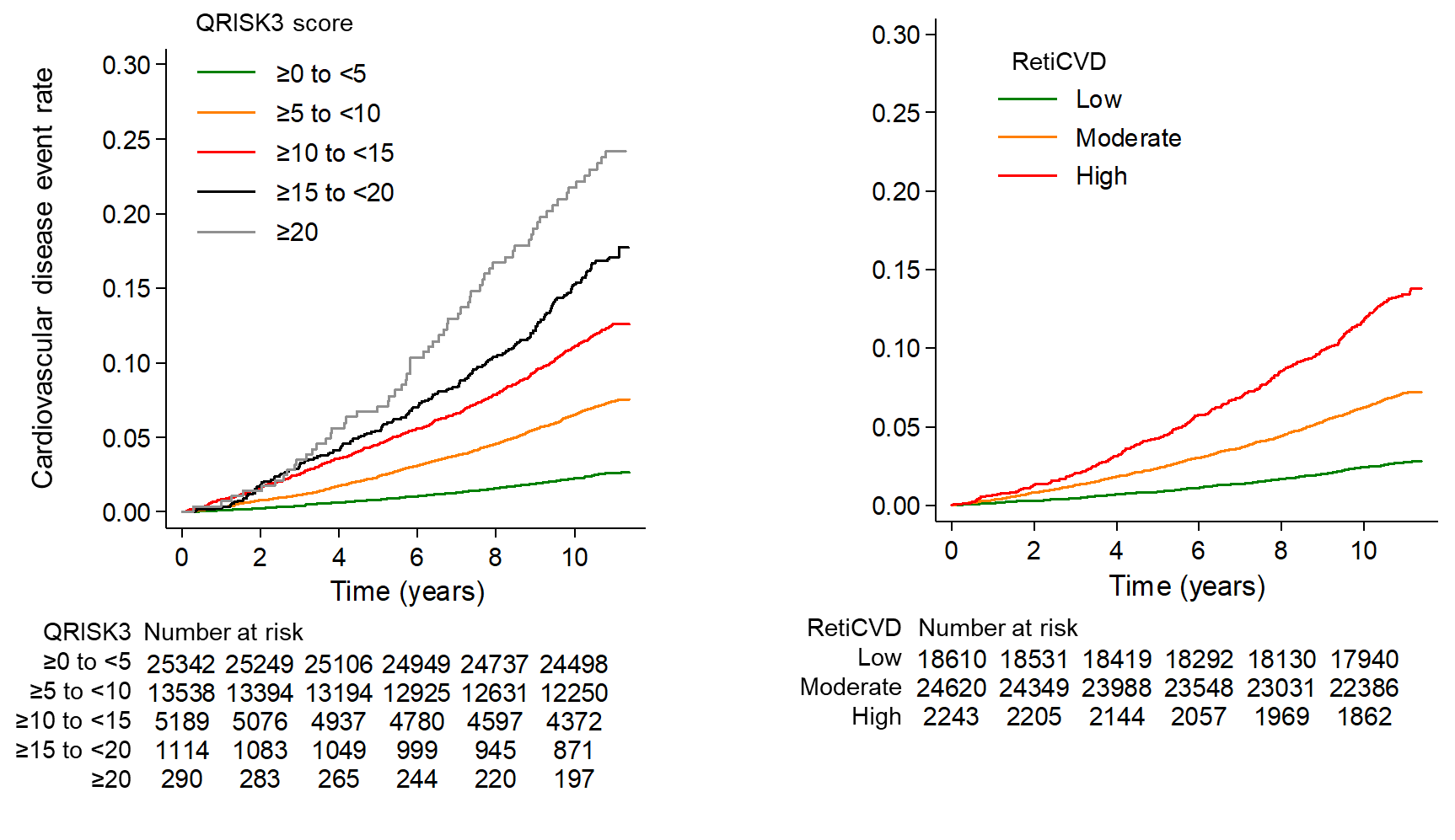


**C QRISK3 in stage 1 hypertension cohort D Reti-CVD in stage 1 hypertension cohort**


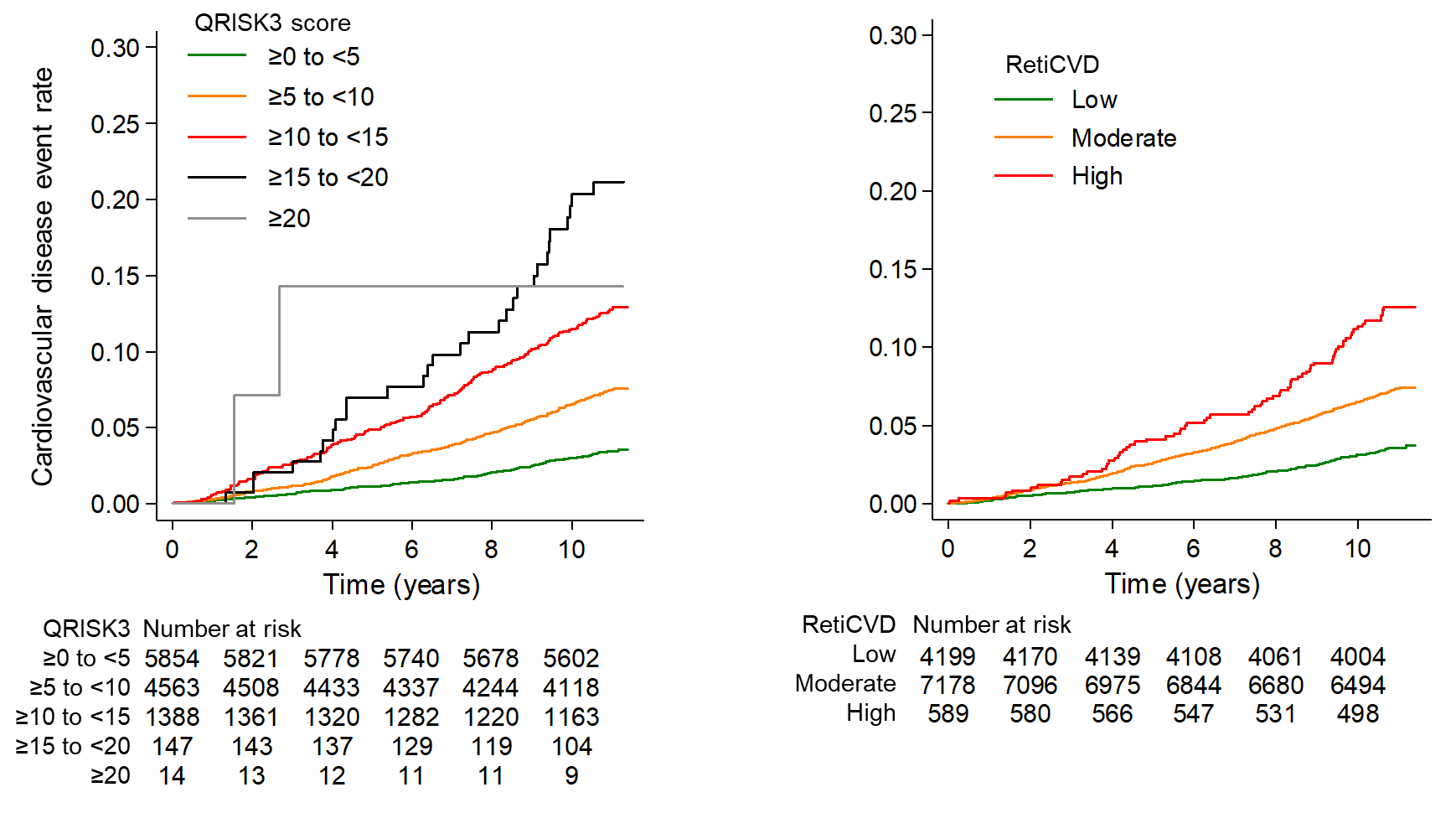

Supplement: Supplementary file 4 — Additional file 4: eFigure 3. Kaplan-Meier curves according to Reti-CVD and QRISK3. [file 12916_2022_2684_MOESM4_ESM.docx]
