# Supplementary material for: Validation of a deep-learning-based retinal biomarker (Reti-CVD) in the prediction of cardiovascular disease: data from UK Biobank
Source: BMC Med. 2023 Jan 24;21:28. doi: 10.1186/s12916-022-02684-8 (PMC9872417; doi:10.1186/s12916-022-02684-8)
Supplement: Supplementary file 5 — Additional file 5: eTable 1. Risk of cardiovascular events by the deep-learning-based retinal CVD biomarker (Reti-CVD). [file 12916_2022_2684_MOESM5_ESM.docx]

## Additional file 5: eTable 1. Risk of cardiovascular events by the deep-learning-based retinal CVD biomarker (Reti-CVD)

| Risk predictor | N | Cases | person-years | Incidence  (95% CI) | | | | | |  | Unadjusted Hazard Ratio (95% CI) | | | | | |  |
| --- | --- | --- | --- | --- | --- | --- | --- | --- | --- | --- | --- | --- | --- | --- | --- | --- | --- |
| **Non-statin Cohort (n=45,473)** | |  |  |  |  |  |  |  |  |  |  |  |  |  |  |  |  |
| Reti-CVD |  |  |  |  |  |  |  |  |  |  |  |  |  |  |  |  |  |
| Low | 18,610 | 504 | 201,847 | 2.5 | ( | 2.3 | - | 2.7 | ) |  | 1 (reference) | | |  |  |  |  |
| Moderate | 24,620 | 1,725 | 259,916 | 6.6 | ( | 6.3 | - | 7.0 | ) |  | 2.67 | ( | 2.42 | - | 2.95 | ) |  |
| High | 2,243 | 293 | 22,786 | 12.9 | ( | 11.5 | - | 14.4 | ) |  | 5.23 | ( | 4.53 | - | 6.04 | ) |  |
|  |  |  |  |  |  |  |  |  |  |  | Adjusted HR trend* | | | | | |  |
|  |  |  |  |  |  |  |  |  |  |  | 1.39 | ( | 1.29 | - | 1.51 | ) |  |
| QRISK score |  |  |  |  |  |  |  |  |  |  |  |  |  |  |  |  |  |
| ≥0 to <5 | 25,342 | 654 | 275,180 | 2.4 | ( | 2.2 | - | 2.6 | ) |  | 1 (reference) | | |  |  |  |  |
| ≥5 to <10 | 13,538 | 988 | 142,767 | 6.9 | ( | 6.5 | - | 7.4 | ) |  | 2.93 | ( | 2.66 | - | 3.24 | ) |  |
| ≥10 to <15 | 5,189 | 631 | 52,838 | 11.9 | ( | 11.0 | - | 12.9 | ) |  | 5.10 | ( | 4.57 | - | 5.69 | ) |  |
| ≥15 to <20 | 1,114 | 184 | 11,052 | 16.6 | ( | 14.4 | - | 19.2 | ) |  | 7.16 | ( | 6.08 | - | 8.44 | ) |  |
| ≥20 | 290 | 65 | 2,712 | 24.0 | ( | 18.8 | - | 30.6 | ) |  | 10.49 | ( | 8.13 | - | 13.54 | ) |  |
|  |  |  |  |  |  |  |  |  |  |  |  |  |  |  |  |  |  |
| Total | 45,473 | 2,522 | 484,549 | 5.2 | ( | 5.0 | - | 5.4 | ) |  |  |  |  |  |  |  |  |
|  |  |  |  |  |  |  |  |  |  |  |  |  |  |  |  |  |  |
| **Stage 1 Hypertension Cohort (n=11,966)** | | | | | | | | | | | | | | | | | |
| Reti-CVD |  |  |  |  |  |  |  |  |  |  |  |  |  |  |  |  |  |
| Low | 4,199 | 149 | 45,360 | 3.3 | ( | 2.8 | - | 3.9 | ) |  | 1 (reference) | | |  |  |  |  |
| Moderate | 7,178 | 520 | 75,629 | 6.9 | ( | 6.3 | - | 7.5 | ) |  | 2.10 | ( | 1.75 | - | 2.52 | ) |  |
| High | 589 | 72 | 6,051 | 11.9 | ( | 9.4 | - | 15.0 | ) |  | 3.66 | ( | 2.77 | - | 4.85 | ) |  |
|  |  |  |  |  |  |  |  |  |  |  | Adjusted HR trend* | | | | | |  |
|  |  |  |  |  |  |  |  |  |  |  | 1.36 | ( | 1.18 | - | 1.58 | ) |  |
| QRISK score** |  |  |  |  |  |  |  |  |  |  |  |  |  |  |  |  |  |
| ≥0 to <5 | 5,854 | 202 | 63,330 | 3.2 | ( | 2.8 | - | 3.7 | ) |  | 1 (reference) | | |  |  |  |  |
| ≥5 to <10 | 4,563 | 335 | 48,042 | 7.0 | ( | 6.3 | - | 7.8 | ) |  | 2.38 | ( | 1.97 | - | 2.86 | ) |  |
| ≥10 to <15 | 1,402 | 175 | 14,255 | 12.3 | ( | 10.6 | - | 14.2 | ) |  | 4.47 | ( | 3.62 | - | 5.52 | ) |  |
| ≥15 | 147 | 29 | 1,413 | 20.5 | ( | 14.3 | - | 29.5 | ) |  | 7.41 | ( | 4.83 | - | 11.36 | ) |  |
|  |  |  |  |  |  |  |  |  |  |  |  |  |  |  |  |  |  |
| Total | 11,966 | 741 | 127,040 | 5.8 | ( | 5.4 | - | 6.3 | ) |  |  |  |  |  |  |  |  |

*Based on multivariable model after adjusting QRISK3 five groups. **For stage 1 hypertension cohort, highest QRISK3 groups was defined as ≥15% due to lack of number of ≥20% group. Incidence per 1000 person-years. CI=confidence interval. CVD=cardiovascular disease. HR=hazard ratio. N=number at risk. Reti-CVD=deep-learning-based retinal CVD biomarker.
