# Supplementary material for: Validation of a deep-learning-based retinal biomarker (Reti-CVD) in the prediction of cardiovascular disease: data from UK Biobank
Source: BMC Med. 2023 Jan 24;21:28. doi: 10.1186/s12916-022-02684-8 (PMC9872417; doi:10.1186/s12916-022-02684-8)
Supplement: Supplementary file 6 — Additional file 6: eTable 2. Subgroup analysis in the UK Biobank. [file 12916_2022_2684_MOESM6_ESM.docx]

## Additional file 6: eTable 2. Subgroup analysis in the UK Biobank

| At-risk Subgroup and Reti-CVD | N | Cases | person-years | Incidence  (95% CI) | | | | | |  | Unadjusted Hazard Ratio (95% CI) | | | | | |
| --- | --- | --- | --- | --- | --- | --- | --- | --- | --- | --- | --- | --- | --- | --- | --- | --- |
| High BMI |  |  |  |  |  |  |  |  |  |  |  |  |  |  |  |  |
| Low | 11,265 | 384 | 121,726 | 3.2 | ( | 2.9 | - | 3.5 | ) |  | 1 (reference) |  |  |  |  |  |
| Moderate | 18,258 | 1,434 | 191,862 | 7.5 | ( | 7.1 | - | 7.9 | ) |  | 2.38 | ( | 2.13 | - | 2.67 | ) |
| High | 1,816 | 266 | 18,334 | 14.5 | ( | 12.9 | - | 16.4 | ) |  | 4.67 | ( | 3.99 | - | 5.46 | ) |
|  |  |  |  |  |  |  |  |  |  |  | Adjusted HR trend* | | | | | |
| Total | 31,339 | 2,084 | 331,922 | 6.3 | ( | 6.0 | - | 6.6 | ) |  | 1.37 | ( | 1.25 | - | 1.49 | ) |
|  |  |  |  |  |  |  |  |  |  |  |  |  |  |  |  |  |
| Participant on antihypertensive medication | | | |  |  |  |  |  |  |  |  |  |  |  |  |  |
| Low | 1,637 | 105 | 17,381 | 6.0 | ( | 5.0 | - | 7.3 | ) |  | 1 (reference) |  |  |  |  |  |
| Moderate | 5,642 | 590 | 58,486 | 10.1 | ( | 9.3 | - | 10.9 | ) |  | 1.68 | ( | 1.36 | - | 2.07 | ) |
| High | 846 | 148 | 8,376 | 17.7 | ( | 15.0 | - | 20.8 | ) |  | 2.98 | ( | 2.32 | - | 3.83 | ) |
|  |  |  |  |  |  |  |  |  |  |  | Adjusted HR trend* | | | | | |
| Total | 8,125 | 843 | 84,243 | 10.0 | ( | 9.4 | - | 10.7 | ) |  | 1.30 | ( | 1.13 | - | 1.49 | ) |
|  |  |  |  |  |  |  |  |  |  |  |  |  |  |  |  |  |
| pre-Diabetes or Diabetes | | |  |  |  |  |  |  |  |  |  |  |  |  |  |  |
| Low | 269 | 17 | 2,864 | 5.9 | ( | 3.7 | - | 9.5 | ) |  | 1 (reference) |  |  |  |  |  |
| Moderate | 677 | 87 | 6,930 | 12.6 | ( | 10.2 | - | 15.5 | ) |  | 2.14 | ( | 1.27 | - | 3.59 | ) |
| High | 86 | 15 | 832 | 18.0 | ( | 10.9 | - | 29.9 | ) |  | 3.10 | ( | 1.55 | - | 6.22 | ) |
|  |  |  |  |  |  |  |  |  |  |  | Adjusted HR trend* | | | | | |
| Total | 1,032 | 119 | 10,625 | 11.2 | ( | 9.4 | - | 13.4 | ) |  | 1.19 | ( | 0.82 | - | 1.73 | ) |

High BMI=BMI≥25kg/m2. *Based on multivariable model after adjusting QRISK3 five groups. Incidence per 1000 person-years. BMI=body mass index. CI=confidence interval. CVD=cardiovascular disease. HR=hazard ratio. N=number at risk. Reti-CVD=deep-learning-based retinal CVD biomarker.
