# Supplementary material for: Validation of a deep-learning-based retinal biomarker (Reti-CVD) in the prediction of cardiovascular disease: data from UK Biobank
Source: BMC Med. 2023 Jan 24;21:28. doi: 10.1186/s12916-022-02684-8 (PMC9872417; doi:10.1186/s12916-022-02684-8)

## Additional file 7: eFigure 4. Decision curve analysis

## **A** In General Population **B** hypertensive patients **C** Pre-Diabetes and Diabetes
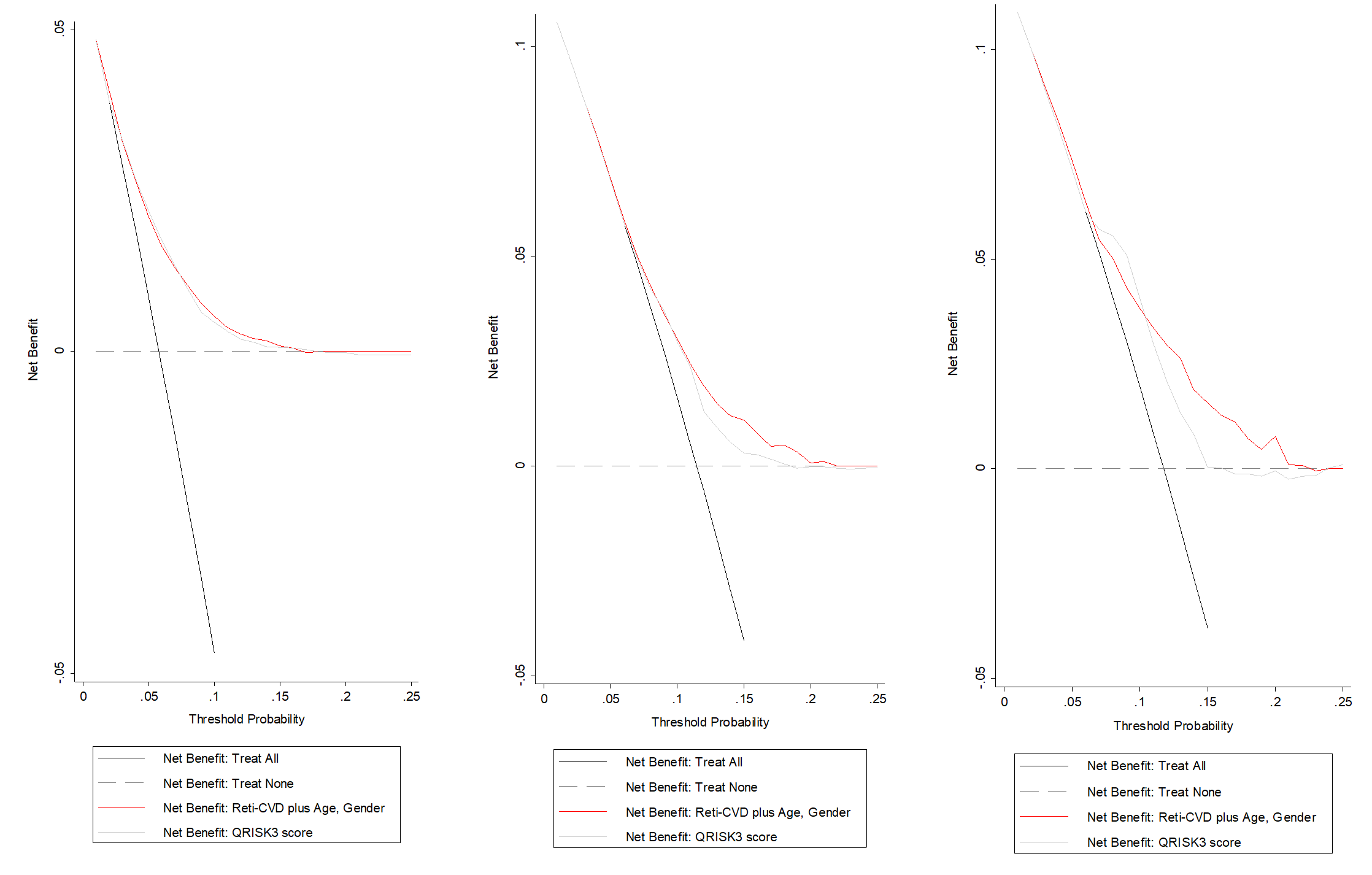

Supplement: Supplementary file 7 — Additional file 7: eFigure 4. Decision curve analysis. [file 12916_2022_2684_MOESM7_ESM.docx]
