# Supplementary material for: Validation of a deep-learning-based retinal biomarker (Reti-CVD) in the prediction of cardiovascular disease: data from UK Biobank
Source: BMC Med. 2023 Jan 24;21:28. doi: 10.1186/s12916-022-02684-8 (PMC9872417; doi:10.1186/s12916-022-02684-8)
Supplement: Supplementary file 8 — Additional file 8: eTable 3. Detailed distribution of Reti-CVD risk groups among those between 7.5% and 10% QRISK3 score. [file 12916_2022_2684_MOESM8_ESM.docx]

## Additional file 8: eTable 3. Detailed distribution of Reti-CVD risk groups among those between 7.5% and 10% QRISK3 score.

| Reti-CVD | ≥7.5 to <8.0 | ≥8.0 to <8.5 | ≥8.5 to <9.0 | ≥9.0 to <9.5 | ≥9.5 to <10.0 | Total |
| --- | --- | --- | --- | --- | --- | --- |
| Low risk | 219 | 157 | 147 | 115 | 96 | 734 |
|  | (3.5) | (2.5) | (2.3) | (1.8) | (1.5) | (11.7) |
| Moderate risk | 1,160 | 1,078 | 956 | 969 | 828 | 4,991 |
|  | (18.4) | (17.1) | (15.2) | (15.4) | (13.2) | (79.3) |
| High risk | 103 | 118 | 115 | 121 | 112 | 569 |
|  | (1.6) | (1.9) | (1.8) | (1.9) | (1.8) | (9.0) |
|  |  |  |  |  |  |  |
| Total | 1,482 | 1,353 | 1,218 | 1,205 | 1,036 |  |
|  | (23.6) | (21.5) | (19.4) | (19.2) | (16.5) |  |
